# Assessing Sports‐Related Concussion in Aboriginal and Torres Strait Islander Peoples: First Responder and Health‐Care Workers Feedback on the Sport Concussion Assessment Tool

**DOI:** 10.1002/hpja.70053

**Published:** 2025-05-13

**Authors:** Kahlia McCausland, Elizabeth Thomas, Trish Hill‐Wall, Richard Norman, Jonathan Bullen, Gill Cowen

**Affiliations:** ^1^ Curtin School of Population Health Curtin University Bentley Australia; ^2^ Centre for Clinical Research Excellence Curtin University Bentley Australia; ^3^ Division of Surgery, Faculty of Health & Medical Sciences The University of Western Australia Perth Australia; ^4^ Curtin Graduate Research School Curtin University Bentley Australia; ^5^ Curtin School of Population Health and Curtin enABle Institute Curtin University Bentley Australia; ^6^ Office of the Vice Chancellor Griffith University Nathan Australia; ^7^ Curtin Medical School, Curtin School of Population Health and Curtin Medical Research Institute Curtin University Bentley Australia

**Keywords:** aboriginal and Torres Strait Islander peoples, concussion, indigenous Australians, mild traumatic brain injury, sport‐related concussion

## Abstract

Sport‐related concussion is a rising public health concern with claims that there is a concussion crisis in sport. An estimated 36% of Aboriginal and Torres Strait Islander peoples participate in sport‐related activity at least once per week; yet, there is a paucity of information relating to concussion assessment in this population. We present and discuss initial findings from research topic yarning about the Sport‐related Concussion Assessment Tool (5th edition) with 25 Aboriginal peoples trained in primary health care or with healthcare or first responder role experience, with the aim of promoting discussion as to how best to ensure Aboriginal and Torres Strait Islander peoples receive appropriate assessment after sustaining a sport‐related concussion.

1

Sport‐related concussion is a rising public health concern with claims that there is a concussion crisis in sport [1]. An estimated 36% of Aboriginal and Torres Strait Islander peoples (age 18+) participate in sport‐related activity at least once per week [2], and Aboriginal and Torres Strait Islander men are overrepresented in boxing, rugby league (14% of National Rugby League players [3]) and Australian rules football (8% of Australian Football League listed players [4]) [5]. Despite this, there is little literature related to concussion among this population. The Sport Concussion Assessment Tool (SCAT) is the standardised tool for evaluating concussions designed for use by physicians and healthcare professionals [6], but it is unclear if this tool is appropriate for use with Aboriginal and Torres Strait Islander peoples [7]. Various versions of the SCAT have been translated internationally, and translation evaluation of the validity and reliability has been studied [8, 9], but there are over 250 Indigenous languages of Australia making such translation a complex task. This letter discusses initial findings from research topic yarning about the SCAT5 (5th edition) with 25 Aboriginal peoples trained in primary health care or with healthcare or first responder role experience [10]. We hope that this letter stimulates discussion as to how best to ensure Aboriginal and Torres Strait Islander peoples receive appropriate assessment after sustaining a sport‐related concussion.

Data collection included four face‐to‐face individual yarns and two group yarns. Participants were shown the SCAT5 proforma and asked to provide general comments on its appearance and content. Participants reported that the format was too busy, the font size was too small, the sentences were too lengthy to understand, and the language was not appropriate with poor choices of word use and a need for simplification of dialogue.

One participant stated, ‘this is unsuitable for Aboriginal people’ reporting that the symptoms of concussion were poorly communicated for Aboriginal peoples' understanding. Another identified that the tool assumed that ‘the athlete is literate, [and] that English is the first language’. Key themes included the need for use of pictures, symbols, diagrams and visual prompts to assist with understanding and communication of symptoms with medical staff with one participant stating, ‘can I suggest drawings and pictures for communication’. Furthermore, it was identified that symbols and shapes may be preferred for recall testing. One participant reported, ‘[the SCAT5] should have diagrams and symbols/shapes, not numbers or words’, and another identified neurodiverse individuals may ‘struggle with this [SCAT5 assessment tool] even without an injury’.

With regards specifically to the symptom checklist, participants felt that the type was too small, simpler language including pictures was needed and that it should be rephrased in terms of what ‘young athletes would say and/or describe’. One participant suggested that further context was required, for example instead of scoring ‘sadness’ asking ‘do you feel unusually sad?’

Whilst the SCAT5 has been superseded since this data collection [11], we believe that our preliminary findings remain relevant to the SCAT6 where, for example, Step 2: Symptom Evaluation, continues to require the athlete to read, select and score their symptoms and Step 3: Cognitive Screening, which is based on the Standardised Assessment of Concussion (SAC), which is to our knowledge is not validated with Aboriginal and Torres Strait Islander peoples. Instructions remain lengthy, for example, ‘I am going to read a string of numbers and when I am done, you repeat them back to me in reverse order of how I read them to you’, and despite a clearer format, there remains no pictorial or visual prompts.

The lack of comprehensive sport‐related concussion data in Aboriginal and Torres Strait Islander peoples itself hinders targeted research into sport‐related concussion assessment in those that need it most. We have engaged in further work collecting feedback from Aboriginal and Torres Strait Islander peoples *without* healthcare or first response experience [12], which we hope will further inform concussion assessment tools in this population in Western Australia. Validity of the SCAT6 in specific populations of Aboriginal or Torres Strait Islander peoples may be of value, but it must not be assumed that a tool is valid for all Mobs, Clans, language groups or dialects. A large study of Aboriginal and Torres Strait Islander peoples engaged in both recreational sports and professional sports, including Aboriginal and Torres Strait Islander peoples whose first language is English and those whose first language is an identified Indigenous Australian language, and non‐Indigenous Australians with English first language would be required. Such a task would involve translators proficient in English and Indigenous Australian languages being researched to allow for initial translation, integration of translations, back‐translation, expert evaluation and review, pre‐testing, finalisation and test–retest reliability. Research to date is insufficient to provide adequate support for such a study, and further Aboriginal and Torres Strait Islander‐led yarning, collaboration and data analysis is needed to ensure research funding is used appropriately and wisely in the quest to ensure best practice concussion assessment in Aboriginal and Torres Strait Islander peoples.

We encourage further Aboriginal and Torres Strait Islander peoples‐led concussion research including researchers, relevant stakeholders and people with lived experience. There is significant scope for this work to be extended to establish a validated concussion assessment tool that is inclusive of, applicable to, and relevant to Aboriginal and Torres Strait Islander peoples.

## Ethics Statement

Ethics approval was obtained from the Western Australian Aboriginal Health Ethics Committee (HREC1012) and reciprocal ethics approval from Curtin University (HRE2020‐0690).

## Consent

The authors have nothing to report.

## Conflicts of Interest

Dr. Cowen is a founding member of the Western Australian Concussion Network, a member of Connectivity TBI Australia and sits on the Community and Regional Football Committee of the Western Australian Football Commission. She currently sits on the MRFF Traumatic Brain Injury Expert Advisory Panel. The authors have no other competing interests to disclose.

## Data Availability

The data that support the findings of this study are available from the corresponding author upon reasonable request.
